# Dataset of China's non-competitive constant price input-output tables for 2007 and 2012

**DOI:** 10.1016/j.dib.2019.104760

**Published:** 2019-11-07

**Authors:** Miao Yu, Xintong Zhao, Yuning Gao

**Affiliations:** a School of Public Policy and Management, Tsinghua University, China; b School of Labor and Human Resources, Renmin University of China, China

**Keywords:** Input-output table, Sector adjustment, Price deflator, Electricity consumption

## Abstract

The China's Input-Output tables for 2007 and 2012 which were published by the China National Bureau of Statistics are competitive current price input-output tables. Based on these tables, this paper constructs the China's non-competitive constant price input-output data for 2007 and 2012. This dataset is supplementary to Ref. [1]. And we share the raw data about China's IO tables for 2007 and 2012. Furthermore, the new IO tables data which we constructed will also be uploaded.

**Table:** Specifications Table

Subject	economics
Specific subject area	input-output analysis, energy economics
Type of data	Table(Excel)
How data were acquired	Collected and calculated based on open statistical data
Data format	Raw, Analyzed
Parameters for data collection	the sectoral outputs ($x_{d}$), the final demands ($f_{d}$), the imports ($m_{d}$), the errors ($e_{d}$), the value-added vector ($v_{d}^{\text{'}}$), the price index in sector *i* in year 2007($P_{2007}^{i}$); the price index in sector *i* in year 2012($P_{2012}^{i}$)
Description of data collection	Open data from the China Statistical Yearbook, China National Bureau of Statistics
Data source location	Industrial sector level in China
Data accessibility	Input data: the China Statistical Yearbook, China National Bureau of Statistics(http://data.stats.gov.cn/english/easyquery.htm?cn=C01), China's IO tables for 2007 and 2012(http://www.stats.gov.cn/ztjc/tjzdgg/trccxh/zlxz/trccb/201701/t20170113_1453448.html) Output data: The China's non-competitive constant price input-output tables for 2007 and 2012 can be found as the excel spreadsheet “new IO2007” and “new IO2012” upon decompressing the supplemental file “data.zip”.
Related research article	Yu, M., Zhao, X., & Gao, Y. 2019. Factor Decomposition of China's Industrial Electricity Consumption Using Structural Decomposition Analysis. Structural Change and Economic Dynamics, 51,67–76. DOI: http://doi.org/10.1016/j.strueco.2019.08.002.

**Table:** 

**Value of the Data** • China's IO tables published by the China National Bureau of Statistics are competitive current price input-output tables, which are not suitable for cross-year input-output analysis. This paper builds the China's non-competitive constant price input-output data. • The China's non-competitive constant price input-output data for 2007 and 2012 constructed in this paper is useful for industrial association analysis, energy input-output analysis, evaluating the drivers of greenhouse gas and pollutant emission changes caused by energy consumption. • The methodology reported in this paper can facilitate construction of the long-term China's non-competitive constant price input-output table data for other years. The data reported here are a complete example of applying that methodology.

## 1 Data

In this paper, China's IO tables for 2007 and 2012 [2], which were published by the National Bureau of Statistics in 2009 and 2015, respectively, are used as supplementary to Ref. [1].

The China's non-competitive constant price input-output tables for 2007 and 2012 can be found as the excel spreadsheet “new IO2007” and “new IO2012” upon decompressing the supplemental file “data.zip”.

Besides that, China's electricity consumption data for various industrial sectors and China's price indices data is used.

## 2 Experimental design, materials, and methods

The following four steps are used to process the data in this article. The first step is to adjust the sector divisions used in the 2012 IO table (IO2012) based on the 2007 IO table (IO2007). In order to ensure the consistency of the sectors in the IO2007 and IO2012, some sectors have been merged and 40 sectors are retained. The second step is to useRAS method to adjust IO2007 from the current price to the 2012 price. Because there are only 30 sectors that have electricity consumption data. The third step is to merge the 40 sectors of IO2007 and IO2012 to the 30 sectors. The fourth step is to change the competitive IO2007 and IO2012 to non-competitive IO tables.

We use electricity consumption data for various industrial sectors published by the National Bureau of Statistics in 2007 and 2012. In addition, we also use China's IO tables for 2007 and 2012, which were published by the National Bureau of Statistics in 2009 and 2015, respectively. The sector divisions used in the two IO tables are inconsistent, which is shown in Table 1. In IO2007, “Transport, Storage” and “Post” are merged to “Transport, Storage and Post”. “Scientific Research and Development” and “Technical Services” are merged to “Scientific Research and Development, Technical Services”. There are 40 sectors in the new IO2007. Then the sectors in the IO2012 are adjusted based on this new IO2007. There are also 40 sectors in the new IO2012 (see Table 2, Table 3, Table 4, Table 5, Table 6, Table 7, Table 8).

**Table 1:** Sector classification of IO2007 and IO2012.

Code	2007 IO table	2012 IO table
1	Agriculture, Forestry, Animal Husbandry & Fishery	Farming, Forestry, Animal Production and Fishery
2	Mining and Washing of Coal	Mining and Washing of Coal
3	Extraction of Petroleum and Natural Gas	Extraction of Crude Petroleum and Natural Gas
4	Mining of Metal Ores	Mining of Metal Ores
5	Mining and Processing of Nonmetal Ores and Other Ores	Mining and Quarrying of Nonmetallic Mineral and Other Mineral
6	Manufacture of Foods and Tobacco	Manufacture of Food and Tobacco
7	Manufacture of Textile	Manufacture of Textiles
8	Manufacture of Textile Wearing Apparel, Footwear, Caps, Leather, Fur, Feather and Its products	Manufacture of Textile Wearing Apparel, Footwear, Leather, Fur, Feather and Its Products
9	Processing of Timbers and Manufacture of Furniture	Processing of Timbers and Manufacture of Furniture
10	Papermaking, Printing and Manufacture of Articles for Culture, Education and Sports Activities	Papermaking, Printing and Manufacture of Articles for Culture, Education and Sports Activities
11	Processing of Petroleum, Coking, Processing of Nuclear Fuel	Manufacture of Refined Petroleum, Coke Products, Processing of Nuclear Fuel
12	Chemical Industry	Manufacture of Chemicals and Chemical Products
13	Manufacture of Nonmetallic Mineral Products	Manufacture of Nonmetallic Mineral Products
14	Smelting and Rolling of Metals	Manufacture and Processing of Metals
15	Manufacture of Metal Products	Manufacture of Fabricated Metal Products, Except Machinery and Equipment
16	Manufacture of General Purpose and Special Purpose Machinery	Manufacture of General Purpose Machinery
17	Manufacture of Transport Equipment	Manufacture of Special Purpose Machinery
18	Manufacture of Electrical Machinery and Equipment	Manufacture of Transport Equipment
19	Manufacture of Communication Equipment, Computer and Other Electronic Equipment	Manufacture of Electrical Machinery and Apparatus
20	Manufacture of Measuring Instrument and Machinery for Cultural Activity & Office Work	Manufacture of Communication Equipment, Computer and Other Electronic Equipment
21	Manufacture of Artwork, Other Manufacture	Manufacture of Measuring Instruments
22	Scrap and Waste	Other Manufacture
23	Production and Supply of Electric Power and Heat Power	Scrap and Waste
24	Production and Distribution of Gas	Repair of Fabricated Metal Products, Machinery and Equipment
25	Production and Distribution of Water	Production and Supply of Electricity and Steam
26	Construction	Production and Distribution of Gas
27	Transport, Storage	Production and Distribution of Water
28	Post	Construction
29	Information Transmission, Computer Services and Software	Wholesale and Retail Trade
30	Wholesale and Retail Trades	Transport, Storage and Post
31	Hotels and Catering Services	Accommodation, Food and Beverage Services
32	Financial Intermediation	Information Transmission, Software and Information Technology Services
33	Real Estate	Finance
34	Renting and Leasing, Business Services	Real Estate
35	Scientific Research and Development	Renting and Leasing, Business Services
36	Technical Services	Scientific Research and Development, Technical Services
37	Management of Water Conservancy, Environment and Public Facilities	Management of Water Conservancy, Environment and Public Facilities
38	Services to Households and Other Services	Services to Households, Repair and Other Services
39	Education	Education
40	Health, Social Security and Social Welfare	Health Care and Social Work Activities
41	Culture, Sports and Entertainment	Culture, Sports and Entertainment
42	Public Management and Social Organization	Public Management, Social Security and Social Organization

**Table 2:** Adjustment the row vector of “Papermaking, Printing and Manufacture of Articles for Culture, Education and Sports Activities” sector.

	Industry A_1_	A_2_	…	A_40_	…	Total Output
⋮						
(Manufacture of Artwork) Other Manufacture	↑ 24.11% X_1_	↑24.11%X_2_	…	↑ 24.11%X_40_		
New Papermaking, Printing and Manufacture of Articles for Culture, Education and Sports Activities	→75.89%X_1_	→ 75.89%X_2_	…	→75.89%X_40_		
⋮						
Papermaking, Printing and Manufacture of Articles for Culture, Education and Sports Activities	X_1_	X_2_	…	X_40_		
Value added						
Total Output						

Note: “↑” indicates increment, and “→” indicates final value.

**Table 3:**
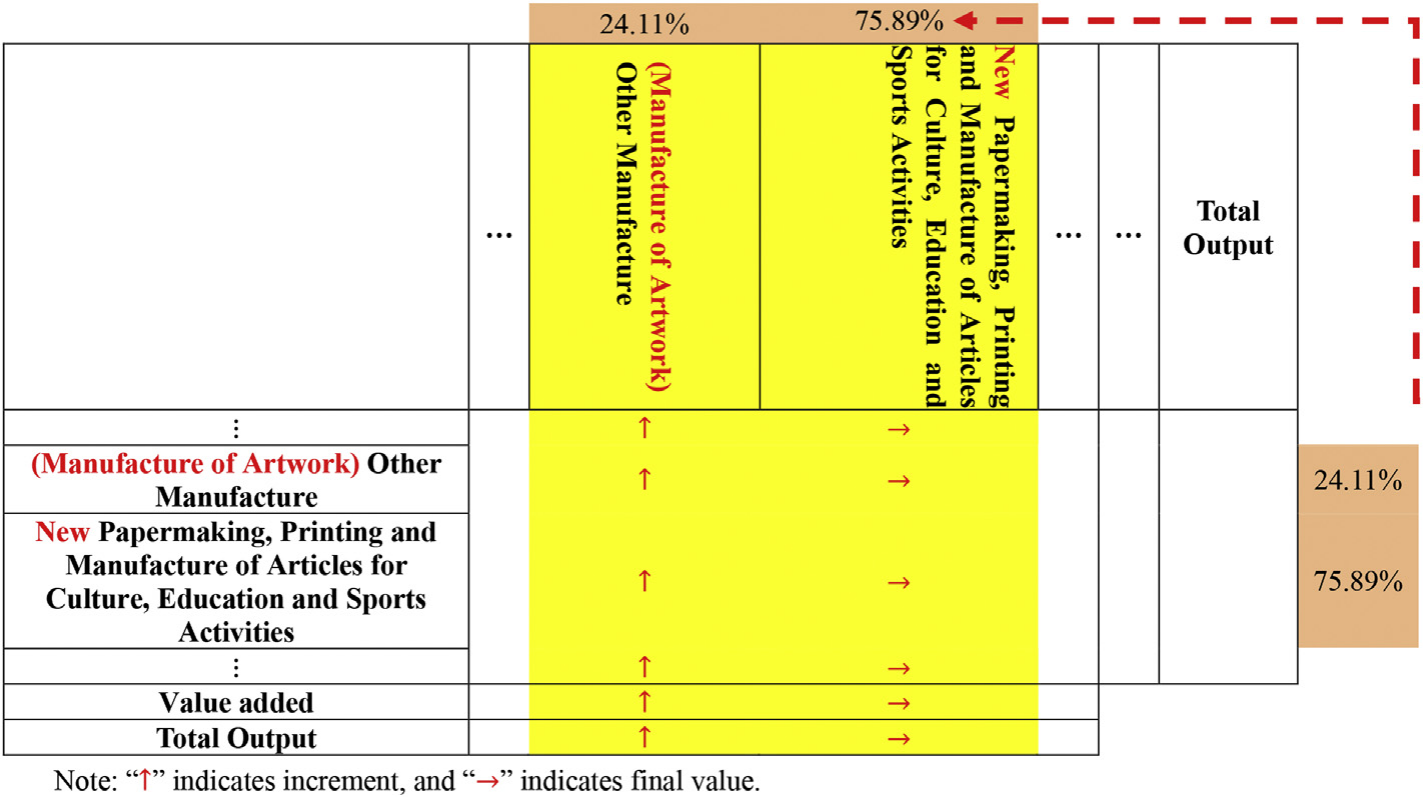
Adjustment the column vector of “Papermaking, Printing and Manufacture of Articles for Culture, Education and Sports Activities" sector.

**Table 4:** The distribution ratio for “Repair of Fabricated Metal Products, Machinery and Equipment” sector.

Industry	Capital Formation(ten thousand yuan)	Ratios of Capital Formation	Errors	Ratios of Errors
Manufacture of Fabricated Metal Products, Except Machinery and Equipment	29338047.57	3.96%	−451902.6	−9.0%
Manufacture of General Purpose and Special Purpose Machinery	338654875.7	45.74%	2215443.7	44.1%
Manufacture of Transport Equipment	271622619.2	36.69%	2384374.7	47.4%
Manufacture of Electrical Machinery and Apparatus	87350294.17	11.80%	409123.0	8.1%
Manufacture of Measuring Instruments	11903422.64	1.61%	5773.6	0.1%
Manufacture of Artwork, Other Manufacture	1505483.174	0.20%	464888.7	9.2%
Total	740374742.4	1	5027701.1	1

**Table 5:** Adjustment the intermediate transactions of “Repair of Fabricated Metal Products, Machinery and Equipment” sector.

	Industry A_1_	A_2_	…	A_40_	…	Total Output
⋮						
Manufacture of Fabricated Metal Products, Except Machinery and Equipment	↑ 3.96% X_1_	↑ 3.96% X_2_	…	↑ 3.96% X_40_		
Manufacture of General Purpose and Special Purpose Machinery	↑ 45.74% X_1_	↑ 45.74% X_2_	…	↑ 45.74% X_40_		
Manufacture of Transport Equipment	↑ 36.69% X_1_	↑ 36.69% X_2_	…	↑ 36.69% X_40_		
Manufacture of Electrical Machinery and Apparatus	↑ 11.80% X_1_	↑ 11.80% X_2_	…	↑ 11.80% X_40_		
Manufacture of Measuring Instruments	↑ 1.61% X_1_	↑ 1.61% X_2_	…	↑ 1.61% X_40_		
Manufacture of Artwork, Other Manufacture	↑ 0.20% X_1_	↑ 0.20% X_2_	…	↑ 0.20% X_40_		
⋮						
Repair of Fabricated Metal Products, Machinery and Equipment	X_1_	X_2_	…	X_40_		
Value added						
Total Output						

Note: “↑” indicates increment.

**Table 6:** Adjustment the errors of “Repair of Fabricated Metal Products, Machinery and Equipment” sector.

	Industry A_1_	A_2_	…	A_40_	…	Errors	Total Output
⋮							
Manufacture of Fabricated Metal Products, Except Machinery and Equipment						↑ −9.0% Y	
Manufacture of General Purpose and Special Purpose Machinery						↑ 44.1% Y	
Manufacture of Transport Equipment						↑ 47.4% Y	
Manufacture of Electrical Machinery and Apparatus						↑ 8.1% Y	
Manufacture of Measuring Instruments						↑ 0.1% Y	
Manufacture of Artwork, Other Manufacture						↑ 9.2%Y	
⋮							
Repair of Fabricated Metal Products, Machinery and Equipment						Y	
Value added							
Total Output							

Note: “↑” indicates increment.

**Table 7:** Adjustment the total output of “Repair of Fabricated Metal Products, Machinery and Equipment” sector.

	…	…	Total Output	Total Output ratio
⋮				
Manufacture of Fabricated Metal Products, Except Machinery and Equipment			$↑\;3.96\text{\%}\sum_{1}^{40}X_{i}$−9.0% Y = 400661.8	4.249%
Manufacture of General Purpose and Special Purpose Machinery			$↑\;45.74\text{\%}\sum_{1}^{40}X_{i}$+44.1% Y = 4316898.8	45.778%
Manufacture of Transport Equipment			$↑\;36.69\text{\%}\sum_{1}^{40}X_{i}$+47.4% Y = 3437249.5	36.450%
Manufacture of Electrical Machinery and Apparatus			$↑\;11.80\text{\%}\sum_{1}^{40}X_{i}$+8.1% Y = 1120198.2	11.879%
Manufacture of Measuring Instruments			$↑\;1.61\text{\%}\sum_{1}^{40}X_{i}$+0.1% Y = 154723.4	1.641%
Manufacture of Artwork, Other Manufacture			$↑\;0.20\text{\%}\sum_{1}^{40}X_{i}$+9.2%Y = 331.1	0.004%
⋮				
Repair of Fabricated Metal Products, Machinery and Equipment				
Value added				
Total Output				

Note: “↑” indicates increment.

**Table 8:**
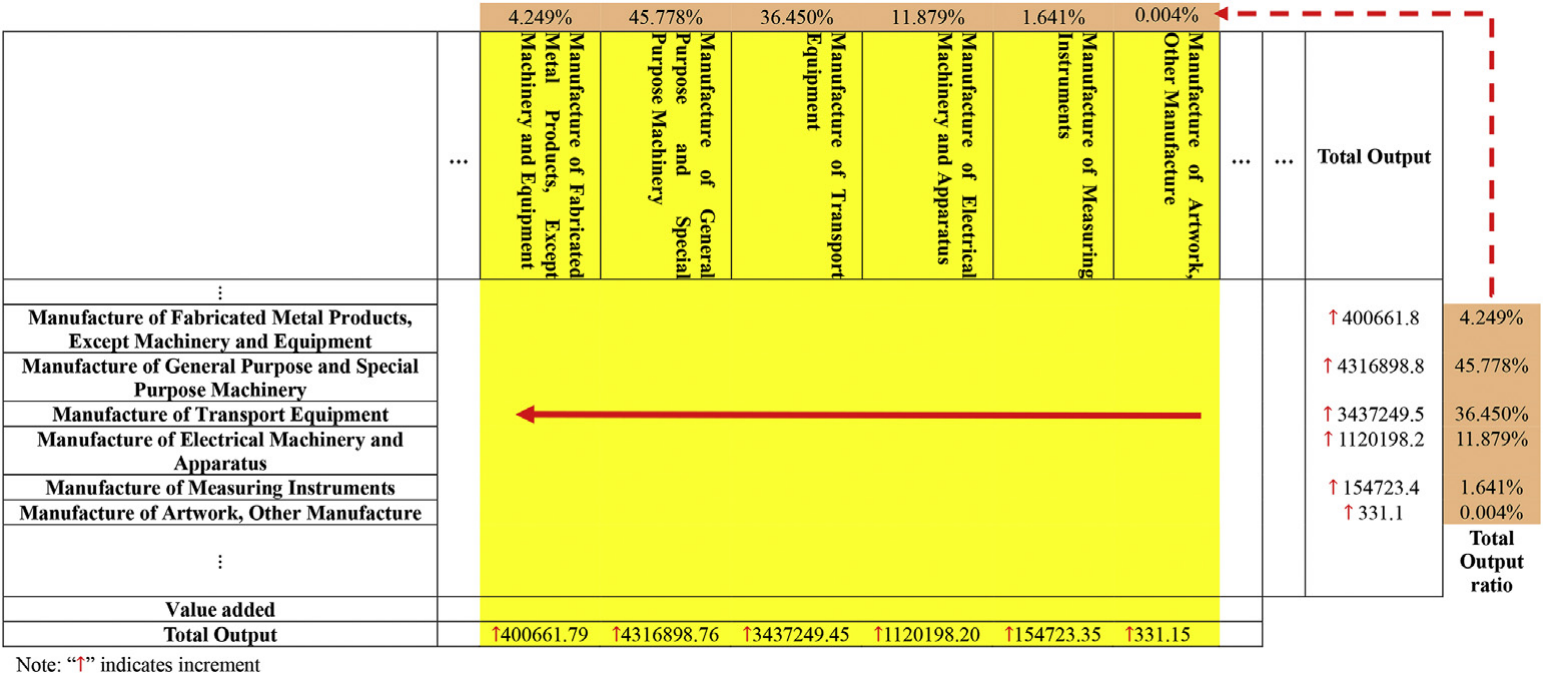
Adjustment the column vector of “Repair of Fabricated Metal Products, Machinery and Equipment” sector.

### 2.1 Sector adjustment of IO2012

We use the departmental consolidation method of constructing the IDE-JETRO International Input Output table used by Meng et al. (2013) [3]and adjusts the divisions used in the IO2012 based on the IO2007.

#### 2.1.1 Manufacture of Artwork, Other Manufacture

##### 2.1.1.1 Current presentation

There are “Manufacture of Artwork, Other Manufacture” sector in the IO2007 and “Other Manufacture” sector in the IO2012. These two sectors are different. It's because that “Manufacture of Artwork” sector is divided into “Papermaking, Printing and Manufacture of Articles for Culture, Education and Sports Activities” sector in IO2012. So “Papermaking, Printing and Manufacture of Articles for Culture, Education and Sports Activities” sector is also different between the IO2007 and IO2012.

##### 2.1.1.2 Adjustment procedure

(1) With the help of the China Industry Statistical Yearbook 2013, we could find the industrial sales output value of “Manufacture of Artwork” sector (655.033 billion yuan) and “Papermaking, Printing and Manufacture of Articles for Culture, Education and Sports Activities” sector (2716.915 billion yuan) in 2012. The percentage of “Manufacture of Artwork” sector in “Papermaking, Printing and Manufacture of Articles for Culture, Education and Sports Activities” sector was 24.11% in 2012.
(2) Using the ratio thus derived, the row vector of “Papermaking, Printing and Manufacture of Articles for Culture, Education and Sports Activities” sector is expanded to a matrix for intermediate transactions.
(3) This ratio is also applied to demarcating the column vector of “Papermaking, Printing and Manufacture of Articles for Culture, Education and Sports Activities” sector.
(4) The “Papermaking, Printing and Manufacture of Articles for Culture, Education and Sports Activities” thus derived is added on to the table.
(5) The row and column vectors of “Papermaking, Printing and Manufacture of Articles for Culture, Education and Sports Activities” are changed to a new one which excluded “Manufacture of Artwork” sector. And “Other Manufacture” sector added “Manufacture of Artwork” sector to form “Manufacture of Artwork, Other Manufacture” sector.

#### 2.1.2 Repair of Fabricated Metal Products, Machinery and Equipment

##### 2.1.2.1 Current presentation

There is standalone “Repair of Fabricated Metal Products, Machinery and Equipment” sector in the IO2012.

##### 2.1.2.2 Adjustment procedure

(1) With the help of the Chinese Standard Industrial Classification (GB/T 4754–2011), the machines list under the “Repair of Fabricated Metal Products, Machinery and Equipment” sector are assumed to be repaired. The industries of these machines include: “Manufacture of Fabricated Metal Products, Except Machinery and Equipment”; “Manufacture of General Purpose and Special Purpose Machinery”; “Manufacture of Transport Equipment”; “Manufacture of Electrical Machinery and Apparatus”; “Manufacture of Measuring Instruments”; “Manufacture of Artwork, Other Manufacture”.
(2) For the commodities identified in (1), the distribution ratio for each industry (column) is derived from the Capital Formation Matrix*1, at the level of grouping permitted by the data's classification.
(3) Using the ratios thus derived, the row vector of “Repair of Fabricated Metal Products, Machinery and Equipment” sector is expanded to a matrix for intermediate transactions.
(4) There are entries at the intersection of “Repair of Fabricated Metal Products, Machinery and Equipment” and Errors, the ratios are derived with respect to Errors. Using the ratios thus derived, the entries are distributed along the Errors. Entries at the intersection of “Repair of Fabricated Metal Products, Machinery and Equipment” and domestic transactions and import matrix are zero.
(5) The sums of increased values are calculated row-wise, which form the total output of “Repair of Fabricated Metal Products, Machinery and Equipment” activity for each type of machinery. The total output ratios are calculated, which are then applied to demarcating the column vector of “Repair of Fabricated Metal Products, Machinery and Equipment” sector.
(6) The “Repair of Fabricated Metal Products, Machinery and Equipment matrix” thus derived is added on to the table.
(7) The row and column vectors of “Repair of Fabricated Metal Products, Machinery and Equipment” are deleted.

#### 2.1.3 Sector classifications

After sector adjustment of IO2012, the sector divisions of IO2007 and IO2012 IO are the same which includes 40 sectors. Table 9 shows the sector classification.

**Table 9:** Sector classification.

Code	Sector name (Chinese IO table)	Code	Sector name (Chinese IO table)
1	Farming, Forestry, Animal Production and Fishery	21	Manufacture of Artwork, Other Manufacture
2	Mining and Washing of Coal	22	Scrap and Waste
3	Extraction of Crude Petroleum and Natural Gas	23	Production and Supply of Electricity and Steam
4	Mining of Metal Ores	24	Production and Distribution of Gas
5	Mining and Quarrying of Nonmetallic Mineral and Other Mineral	25	Production and Distribution of Water
6	Manufacture of Food and Tobacco	26	Construction
7	Manufacture of Textiles	27	Transport、Storage and Post
8	Manufacture of Textile Wearing Apparel, Footwear, Leather, Fur, Feather and Its Products	28	Information Transmission, Software and Information Technology Services
9	Processing of Timbers and Manufacture of Furniture	29	Wholesale and Retail Trade
10	Papermaking, Printing and Manufacture of Articles for Culture, Education and Sports Activities	30	Accommodation, Food and Beverage Services
11	Manufacture of Refined Petroleum, Coke Products, Processing of Nuclear Fuel	31	Finance
12	Manufacture of Chemicals and Chemical Products	32	Real Estate
13	Manufacture of Nonmetallic Mineral Products	33	Renting and Leasing, Business Services
14	Manufacture and Processing of Metals	34	Scientific Research and Development, Technical Services
15	Manufacture of Fabricated Metal Products, Except Machinery and Equipment	35	Management of Water Conservancy, Environment and Public Facilities
16	Manufacture of General Purpose and Special Purpose Machinery	36	Services to Households, Repair and Other Services
17	Manufacture of Transport Equipment	37	Education
18	Manufacture of Electrical Machinery and Apparatus	38	Health Care and Social Work Activities
19	Manufacture of Communication Equipment, Computer and Other Electronic Equipment	39	Culture, Sports and Entertainment
20	Manufacture of Measuring Instruments	40	Public Management, Social Security and Social Organization

### 2.2 RAS method for deflating Chinese IO table

In order to focus on real rather than nominal changes in our decomposition analysis, the IO table used should be corrected based on constant prices. The method that has been most widely used for the estimation of IO tables in constant prices is Double Deflation (DD) [4]. Though this method is generally accepted, it still involves certain problems which have been reported in Sevaldson (1976), Wolff (1994), and Dietzenbacher and Hoen (1998) [[5], [6], [7]]. The two main problems can be summarized as follows: First, under this method, an entire row in the IO table is deflated using the price index of gross output. This method ignores the practical situation where price indices are likely to be different within a row of intermediate deliveries, since most sectors produce more than one good, and each sector requires a different mix of these goods as an input. Second, the published IO table available to the normal user is already largely aggregated, meaning that the user can only adjust the IO table in constant prices via deflation after aggregation. Therefore, the aggregation error may influence the accuracy of the deflation.

To encountering the above problems, Dietzenbacher and Hoen (1998) propose an alternative method from the user's viewpoint [7]. Under their method, the intermediate deliveries in constant prices are estimated on the basis of intermediate deliveries in current prices, and the row and column sums in constant prices. This estimation precisely satisfies the requirements for applying the RAS method. And this method performs better than DD.

The RAS-procedure is a biproportional projection method that was developed for “updating” a given matrix (say $A_{0}$, not necessarily square), such that the updated matrix (${\tilde{A}}_{1}$) satisfies exogenously given row and column sums. The RAS-method proceeds iteratively. In the first step the rows are adjusted. Each row *i* is multiplied by a scalar $r_{i}$ such that the *i*-th row sum equals the prespecified row sum of $A_{1}$. The resulting matrix after step 1 may be denoted as ${\tilde{A}}_{1}\left(1\right)={\hat{r}}_{1}A_{0}$. In the second step, the columns of ${\tilde{A}}_{1}\left(1\right)$ are adjusted so as to satisfy the column sum requirement. This yield ${\tilde{A}}_{1}\left(2\right)={\tilde{A}}_{1}\left(1\right){\hat{s}}_{2}={\hat{r}}_{1}A_{0}{\hat{s}}_{2}$. It is likely, however, that the row sum requirements are violated. Therefore the rows are adjusted again; ${\tilde{A}}_{1}\left(3\right)={\hat{r}}_{3}{\tilde{A}}_{1}\left(2\right){\hat{s}}_{2}={\hat{r}}_{3}{\hat{r}}_{1}A_{0}{\hat{s}}_{2}$. Next, the columns are adjusted again: ${\tilde{A}}_{1}\left(4\right)={\tilde{A}}_{1}\left(3\right){\hat{s}}_{4}={\hat{r}}_{3}{\hat{r}}_{1}A_{0}{\hat{s}}_{2}{\hat{s}}_{4}$, and so forth. Starting with column adjustments in the first step yields ${\tilde{A}}_{1}\left(4\right)={\hat{r}}_{3}{\hat{r}}_{1}A_{0}{\hat{s}}_{2}{\hat{s}}_{4}$ after the fourth step. It can be shown that under mild conditions the iterative procedure converges. The updated matrix can be written as ${\tilde{A}}_{1}=\hat{r}{\tilde{A}}_{0}\hat{s}$ and does not depend on whether the procedure is started with a row adjustment or with a column adjustment.

The RAS-method has been applied to estimate next year's coefficients matrix ($A_{1}$) on the basis of this year's matrix ($A_{0}$), given next year's row and column sums. In this paper we apply the RAS-procedure to estimate the input-output table in constant prices, on the basis of the table in current prices, given the row and column totals in constant prices.

The input-output table in current prices is given in Table 10, the table in constant prices, using the RAS method, in Table 11.

**Table 10:** 2007 IO table in current prices.

Z	f	m	e	x
$v^{'}$				
$x^{'}$

**Table 11:** Using RAS method to form 2007 IO table in constant prices.

$Z_{d}$	$x_{d}+m_{d}-f_{d}-e_{d}$
$x_{d}^{'}-v_{d}^{'}$	–

The *n* × *n* matrix **Z** denotes the intermediate demand matrix, the vector **f** the final demands (rural household consumption, urban household consumption, government consumption, gross fixed capital formation, changes in inventories and exports), the vector **m** the imports, the vector **e** the errors, **x** denotes the vector with sectoral outputs. $v^{'}$ is a row vector, the elements of which are value added of industrial sectors. In Table 11, the subscript *d* (for deflated) is used to indicate that the corresponding matrices and vector are in constant prices.

In this paper we apply the RAS-procedure to estimate the input-output table in constant prices, on the basis of the table in current prices, given the row and column totals in constant prices. In this method, the sectoral outputs ($x_{d}$), the final demands ($f_{d}$), the imports ($m_{d}$), the errors ($e_{d}$) and the value-added vector ($v_{d}^{\text{'}}$) are required to be known.

The element $\pi_{i}$of the vector πdenotes the price deflator in industrial sector *i*. It is defined as the ratio of the base year price and the current price. $\pi_{i}=\frac{P_{2012}^{i}}{P_{2007}^{i}}$ (2012 price is the base year price). To simplify the calculation process, we assume each industry sector has the same price deflator (Liu Qiyun, Peng Zhilong, 2010). For $x_{d}+m_{d}-f_{d}-e_{d}=\hat{\pi }\left(x+m-f-e\right)$, if price deflator$\pi_{i}$ could be got, $x_{d}+m_{d}-f_{d}-e_{d}$would be computable. And if price deflator of value added could be got, $x_{d}^{'}-v_{d}^{'}$alsocan be derived. Then, the intermediate demand matrix in constant prices ($Z_{d}$) could be estimated by the RAS-method.

### 2.3 Price deflator

#### 2.3.1 Price deflators of industrial sectors

Because producer price is used in China's IO table. Relevant producer price indices are used to calculate price deflator of primary industry and secondary industry sectors.^1^

Using the following formula to calculate the price deflator of primary industry and secondary industry sectors:

where, $\frac{P_{2012}^{i}}{P_{2011}^{i}}$, $\frac{P_{2011}^{i}}{P_{2010}^{i}}$, $\frac{P_{2010}^{i}}{P_{2009}^{i}}$, $\frac{P_{2009}^{i}}{P_{2008}^{i}}$, $\frac{P_{2008}^{i}}{P_{2007}^{i}}$ denote the producer price in sector *i* in year 2012, 2011, 2010, 2009, 2008 (preceding year = 100). Data sources: National Bureau of Statistics of China.

For tertiary industry exclude “Finance” and “Real Estate” sector, we use relevant consumer price indices followLiu Qiyun and Peng Zhilong (2010) [8]. This is because China don't have producer price indices for tertiary industry. The relation between the tertiary industry and the price indices in Table 12. The formulas to calculate the price deflator of these industry sectors are as follows:

$$\pi_{i}=\frac{P_{2012}^{i}}{P_{2007}^{i}}=\frac{P_{2012}^{i}}{P_{2011}^{i}}*\frac{P_{2011}^{i}}{P_{2010}^{i}}*\frac{P_{2010}^{i}}{P_{2009}^{i}}*\frac{P_{2009}^{i}}{P_{2008}^{i}}*\frac{P_{2008}^{i}}{P_{2007}^{i}}\;i=27\text{, }28\text{, ... , }39\text{, }40\text{ and }i\;\neq \;31\text{, }32$$

where, $\frac{P_{2012}^{i}}{P_{2011}^{i}}$, $\frac{P_{2011}^{i}}{P_{2010}^{i}}$, $\frac{P_{2010}^{i}}{P_{2009}^{i}}$, $\frac{P_{2009}^{i}}{P_{2008}^{i}}$, $\frac{P_{2008}^{i}}{P_{2007}^{i}}$ denote the producer price in sector *i* in year 2012, 2011, 2010, 2009, 2008 (preceding year = 100). Data sources: National Bureau of Statistics of China.

**Table 12:** Relationship between tertiary industry and price indices.

Industry Sector	Price Index(preceding year = 100)
Transport、Storage and Post	Consumer Price Indices, Transportation and Communication
Information Transmission, Software and Information Technology Services	Consumer Price Index
Wholesale and Retail Trade	Retail Price Indices
Accommodation, Food and Beverage Services	Consumer Price Indices, Dining Out
Renting and Leasing, Business Services	Consumer Price Index
Scientific Research and Development, Technical Services	Consumer Price Index
Management of Water Conservancy, Environment and Public Facilities	Consumer Price Index
Services to Households, Repair and Other Services	Consumer Price Indices, Household Services and Maintenance and Renovation
Education	Consumer Price Indices, Education
Health Care and Social Work Activities	Consumer Price Indices, Health Care Services
Culture, Sports and Entertainment	Consumer Price Indices, Cultural and Recreational Articles
Public Management, Social Security and Social Organization	Consumer Price Index

For “Finance” sector, we take a weighted average of “Consumer Price Index (preceding year = 100)” and “Price Indices for Investment in Fixed Assets (preceding year = 100)” to produce a composite number, which is price deflator of “Finance” sector. The weights are derived from ratio between household consumption expenditure and total investment in fixed assets in the whole country. Data sources: National Bureau of Statistics of China.

For “Real Estate” sector, we use the following formula to calculate its price deflator [9].

$$\frac{P_{2012}^{32}}{P_{2007}^{32}}=\frac{1}{\frac{P_{2007}^{32}*Q_{2007}^{32}}{P_{2012}^{32}*Q_{2012}^{32}}*\frac{P_{2011}^{32}*Q_{2012}^{32}}{P_{2011}^{32}*Q_{2011}^{32}}*\frac{P_{2010}^{32}*Q_{2011}^{32}}{P_{2010}^{32}*Q_{2010}^{32}}*\frac{P_{2009}^{32}*Q_{2010}^{32}}{P_{2009}^{32}*Q_{2009}^{32}}*\frac{P_{2008}^{32}*Q_{2009}^{32}}{P_{2008}^{32}*Q_{2008}^{32}}*\frac{P_{2007}^{32}*Q_{2008}^{32}}{P_{2007}^{32}*Q_{2007}^{32}}}$$

$P_{2007}^{32}\text{*}Q_{2007}^{32}$:2007 value-added of real estate (at 2007 prices).

$P_{2012}^{32}*Q_{2012}^{32}$: 2012 value-added of real estate (at 2012 prices).

Where $\frac{P_{2011}^{32}*Q_{2012}^{32}}{P_{2011}^{32}*Q_{2011}^{32}}$, $\frac{P_{2010}^{32}*Q_{2011}^{32}}{P_{2010}^{32}*Q_{2010}^{32}}$, $\frac{P_{2009}^{32}*Q_{2010}^{32}}{P_{2009}^{32}*Q_{2009}^{32}}$, $\frac{P_{2008}^{32}*Q_{2009}^{32}}{P_{2008}^{32}*Q_{2008}^{32}}$, $\frac{P_{2007}^{32}*Q_{2008}^{32}}{P_{2007}^{32}*Q_{2007}^{32}}$ denote indices of value-added of real estate (preceding year = 100) in 2012, 2011, 2010, 2009, 2008. Data sources: National Bureau of Statistics of China.

The result price deflators of all the 40 industrial sectors are in Table 13.

**Table 13:** Price deflators of industrial sectors (year 2012 = 100).

Industry Sector	Price Deflator
Farming, Forestry, Animal Production and Fishery	147.8
Mining and Washing of Coal	154.1
Extraction of Crude Petroleum and Natural Gas	137.9
Mining of Metal Ores	120
Mining and Quarrying of Nonmetallic Mineral and Other Mineral	130.3
Manufacture of Food and Tobacco	115.1
Manufacture of Textiles	116.2
Manufacture of Textile Wearing Apparel, Footwear, Leather, Fur, Feather and Its Products	109.9
Processing of Timbers and Manufacture of Furniture	110.5
Papermaking, Printing and Manufacture of Articles for Culture, Education and Sports Activities	106.7
Manufacture of Refined Petroleum, Coke Products, Processing of Nuclear Fuel	150.7
Manufacture of Chemicals and Chemical Products	110.5
Manufacture of Nonmetallic Mineral Products	116.1
Manufacture and Processing of Metals	105.1
Manufacture of Fabricated Metal Products, Except Machinery and Equipment	108.3
Manufacture of General Purpose and Special Purpose Machinery	106.3
Manufacture of Transport Equipment	101.6
Manufacture of Electrical Machinery and Apparatus	99.6
Manufacture of Communication Equipment, Computer and Other Electronic Equipment	88.9
Manufacture of Measuring Instruments	98.5
Manufacture of Artwork, Other Manufacture	116.6
Scrap and Waste	102.6
Production and Supply of Electricity and Steam	112
Production and Distribution of Gas	125
Production and Distribution of Water	117.7
Construction	126.5
Transport, Storage and Post	96.7
Information Transmission, Software and Information Technology Services	117.5
Wholesale and Retail Trade	115.4
Accommodation, Food and Beverage Services	137.3
Finance	118.1
Real Estate	165.8
Renting and Leasing, Business Services	117.5
Scientific Research and Development, Technical Services	117.5
Management of Water Conservancy, Environment and Public Facilities	117.5
Services to Households, Repair and Other Services	149.5
Education	106.7
Health Care and Social Work Activities	103.8
Culture, Sports and Entertainment	107.4
Public Management, Social Security and Social Organization	117.5

#### 2.3.2 Price deflator of value added

The computational process for the value added in current prices is more complex. Firstly, in the same way, the value added deflator $\rho_{j}$ is defined as the price ratio between the base year value added price and the current value added price, for product *j*. We could only get 9 value added prices of industrial sectors. They are “Indices of Value-added of Agriculture, Forestry, Animal Husbandry and Fishery Industries”, “Indices of Value-added of Industry”, “Indices of Value-added of Construction”, “Indices of Value-added of Wholesale and Retail Trades”, “Indices of Value-added of Transport, Storage and Post”, “Indices of Value-added of Hotels and Catering Services”, “Indices of Value-added of Financial Intermediation”, “Indices of Value-added of Real Estate” and “Indices of Value-added of Others”. Among them, “Indices of Value-added of Industry” and “Indices of Value-added of Others” cover 24 and 9 industrial sectors respectively. We use the following formula to calculate value added price deflators [9].

$$\rho_{j}=\frac{P_{2012}^{j}}{P_{2007}^{j}}=\frac{1}{\frac{P_{2007}^{j}*Q_{2007}^{j}}{P_{2012}^{j}*Q_{2012}^{j}}*\frac{P_{2011}^{j}*Q_{2012}^{j}}{P_{2011}^{j}*Q_{2011}^{j}}*\frac{P_{2010}^{j}*Q_{2011}^{j}}{P_{2010}^{j}*Q_{2010}^{j}}*\frac{P_{2009}^{j}*Q_{2010}^{j}}{P_{2009}^{j}*Q_{2009}^{j}}*\frac{P_{2008}^{j}*Q_{2009}^{j}}{P_{2008}^{j}*Q_{2008}^{j}}*\frac{P_{2007}^{j}*Q_{2008}^{j}}{P_{2007}^{j}*Q_{2007}^{j}}}\;j=1\text{, }2\text{, ... , }9$$

$P_{2007}^{j}*Q_{2007}^{j}$: 2007 value-added of industry *j* (at 2007 prices).

$P_{2012}^{j}*Q_{2012}^{j}$: 2012 value-added of industry *j* (at 2012 prices).

Where, $\frac{P_{2011}^{j}*Q_{2012}^{j}}{P_{2011}^{j}*Q_{2011}^{j}}$, $\frac{P_{2010}^{j}*Q_{2011}^{j}}{P_{2010}^{j}*Q_{2010}^{j}}$, $\frac{P_{2009}^{j}*Q_{2010}^{j}}{P_{2009}^{j}*Q_{2009}^{j}}$, $\frac{P_{2008}^{j}*Q_{2009}^{j}}{P_{2008}^{j}*Q_{2008}^{j}}$, $\frac{P_{2007}^{j}*Q_{2008}^{j}}{P_{2007}^{j}*Q_{2007}^{j}}$ are indices of value-added of *j* industry sector in year 2012, 2011, 2010, 2009, 2008 (preceding year = 100). Data sources: National Bureau of Statistics of China.

Then these 9 industries' value added in constant price could be got. But “Industry” and “Other” sectors conclude 24 and 9 sub-classification industries and the value added of these sub-classification industries can't be derived from the calculation progress above.

Secondly, we use the price deflators of these sub-classification industries to calculate their value added in constant price, then calculate their proportion structure. Using the value added of “Industry” and “Other” sectors and the sub-classification industries' value added proportion structure, the value added of these sub-classification industries could be computed. Therefore, all these 40 industries’ value added ($\bar{v_{d}^{\text{'}}}$) can be got. However, $x_{d}^{\text{'}}-\bar{v_{d}^{\text{'}}}\neq x_{d}+m_{d}-f_{d}-e_{d}$.

Thirdly, the final value added vector $v_{d}^{\text{'}}$ is obtain from the balancing equations. That is, the equality of the row sums and the column sums imply $\left(x_{d}^{'}-v_{d}^{'}\right)u=u^{'}\left(x_{d}+m_{d}-f_{d}-e_{d}\right)$. u is 40-element column vector, where all the elements are 1.

So $v_{id}^{'}=\frac{\bar{v_{id}^{'}}}{\sum_{i}^{40}\bar{v_{id}^{'}}}u^{'}\left(x_{d}+m_{d}-f_{d}-e_{d}\right)$, and $v_{d}^{'}$can be derived.

The price deflators of industrial sectors and price deflator of value added are used to adjust 2007 IO table from the current price to the 2012 price.

### 2.4 Final sector classifications

There are only 30 sectors that have electricity consumption data published by the National Bureau of Statistics in 2007 and 2012. However, there are 40 sectors inthe adjusted 2007 and 2012 IO tables. This paper merged the 40 sectors of the adjusted 2007 and 2012 IO tables to the 30 sectors which have electricity consumption data. The final sector classifications are shown in Table 14.

**Table 14:** Final sector classification.

Code	Sector name	Code	Sector name
1	Farming, forestry, animal production and fishery	16	Manufacture of communication equipment, computer and other electronic equipment
2	Mining of metal ores	17	Manufacture of measuring instruments
3	Mining and quarrying of nonmetallic mineral and other mineral	18	Manufacture of artwork, other manufacture
4	Manufacture of food and tobacco	19	Scrap and waste
5	Manufacture of textiles	20	Mining and washing of coal
6	Manufacture of textile wearing apparel, footwear, leather, fur, feather and its products	21	Extraction of crude petroleum and natural gas
7	Processing of timbers and manufacture of furniture	22	Manufacture of refined petroleum, coke products, processing of nuclear fuel
8	Papermaking, printing and manufacture of articles for culture, education and sports activities	23	Production and supply of electricity and steam
9	Manufacture of chemicals and chemical products	24	Production and distribution of gas
10	Manufacture of nonmetallic mineral products	25	Production and distribution of water
11	Manufacture and processing of metals	26	Construction
12	Manufacture of fabricated metal products, except machinery and equipment	27	Transport、storage and post
13	Manufacture of general purpose and special purpose machinery	28	Information transmission, software and information technology services
14	Manufacture of transport equipment	29	Wholesale and retail trade, accommodation, food and beverage services
15	Manufacture of electrical machinery and apparatus	30	Other service industries

## 3 Non-competitive IO tables

There are two assumptions: 1. no re-export trade; 2. sector internal product is homogenous.

The 2007 and 2012 China's IO tables published by the National Bureau of Statistic are competitive which include imports.

M=(m1⋮mn),Z=(z11⋯z1n⋮⋱⋮zn1⋯znn),T=(1∑p=1nz1p⋮1∑p=1nznp),p=1, 2, …, n.

**M** is the *n*th-dimension import column vector, where $m_{j}$ represents the total import of the *j*th department. **Z** is the *n* × *n* competitive intermediate demand matrix. The $z_{ij}$ terms represent interindustry sales by sector *i* (also known as intermediate sales) to all sectors *j* (including itself, when *j* = *i*), and $z_{ij}$ includes imports. $z_{ij}=z_{ij}^{d}+z_{ij}^{m}$, where $z_{ij}^{d}$terms represent interindustry sales from the domestic market and $z_{ij}^{m}$terms represent interindustry sales from overseas market. T is the *n*th-dimension column vector.

The same proportion (mi∑p=1nzip) is used to split $z_{ij}^{m}$from the interindustry sales by sector *i*,then, $z_{ij}^{m}=$(mi∑p=1nzip)$z_{ij}$.

$$Z^{d}=\left(\begin{array}{ccc} z_{11}^{d} & \cdots & z_{1n}^{d}\\ \vdots & \ddots & \vdots \\ z_{n1}^{d} & \cdots & z_{nn}^{d} \end{array}\right)$$

MˆTˆZ=(z11m⋯z1nm⋮⋱⋮zn1m⋯znnm)=((m1∑p=1nz1p)z11⋯(m1∑p=1nz1p)z1n⋮⋱⋮(mn∑p=1nznp)zn1⋯(mn∑p=1nznp)znn)

$Z^{d}$ is the *n* × *n* non-competitive intermediate demand matrix, which are excluded imports. $\hat{M}\hat{T}Z$ is the *n* × *n* intermediate import demand matrix. Therefore, $Z^{d}=Z-\hat{M}\hat{T}Z$.

Through the above data processing process, the China's non-competitive constant price input-output data for 2007 and 2012 could be got.

## Footnotes

1 For “Construction”, there isn't relevant producer price, so we use “Price Indices for Investment in Fixed Assets (preceding year = 100), Construction and Installation” instead.
